# Differential expression of Kir4.1 and aquaporin 4 in the retina from endotoxin-induced uveitis rat

**Published:** 2007-03-01

**Authors:** Xiao-Qiang Liu, Hideyuki Kobayashi, Zi-Bing Jin, Akihiko Wada, Nobuhisa Nao-i

**Affiliations:** 1 Department of Ophthalmology, Faculty of Medicine, University of Miyazaki, Kiyotake, Miyazaki, Japan; 2 Department of Pharmacology, Faculty of Medicine, University of Miyazaki, Kiyotake, Miyazaki, Japan

## Abstract

**Purpose:**

The inwardly rectifying potassium channel protein Kir4.1 and the water channel protein aquaporin-4 (AQP4) have been suggested to play essential roles in the potassium and water homeostasis of the retina. In this study, we investigated the expression of Kir4.1 and AQP4 in the retina during endotoxin-induced uveitis (EIU) in rats.

**Methods:**

EIU was induced in male Wistar rats by intravitreal injection of lipopolysaccharide (LPS). The severity of the EIU was evaluated by clinical and histopathological examination. The expression of Kir4.1 and AQP4 in the retina was detected by semiquantitative reverse transcriptase polymerase chain reaction (RT-PCR), Western blotting, and immunohistochemical staining.

**Results:**

In the animal model of EIU, the clinical changes correlated well with the histopathological findings. The inflammation peaked at 24 h and resolved by seven day. After an intravitreal LPS injection, the expression of Kir4.1 in the retina showed a significant decline at both the protein and mRNA levels. In the early stages of EIU, the expression of Kir4.1 mRNA decreased sharply, reaching a minimum at 12 h (31%, p<0.001). It then increased gradually and had partially recovered 14 days after LPS injection (92%, p>0.05). The expression of Kir4.1 protein decreased significantly, reaching a minimum at three days after the LPS injection (43%, p<0.001). Thereafter, it increased slightly but was maintained at a low level until 14 days after LPS injection (64%, p<0.001). In contrast, the expression of AQP4 mRNA remained almost unchanged after LPS treatment (p>0.05). The expression of AQP4 protein was only slightly reduced at one day (82%, p>0.05) after LPS injection and then increased gradually and had nearly recovered to the basal level at 14 days after LPS injection.

**Conclusions:**

EIU differently alters the expression of Kir4.1 and AQP4 in the retina. The differential expression of Kir4.1 and AQP4 during EIU implies a disturbance of water and potassium transport in the retina, which may contribute to the retinal edema during ocular inflammation.

## Introduction

Retinal edema is characterized by excessive fluid accumulation in the retina (macula) and is considered to be a serious complication of ocular inflammation [1-3]. It is one of the leading causes of visual impairment in patients with uveitis, with an incidence varying between 24% and 52% depending on the etiology [4,5]. Inflammatory retinal edema is considered to occur primarily after the breakdown of the blood-retinal barrier (BRB), which is associated with retinal vascular leakage, resulting in vasogenic edema [3,6,7]. However, other reports have suggested that water accumulation within the retinal Müller cells (cytotoxic edema) may also contribute to macular edema [2,8,9]. The molecular mechanisms and pathophysiology of retinal edema in endotoxin-induced uveitis (EIU) have only been partly clarified.

Recent studies of glial potassium channels and water channels have increased our understanding of the mechanisms of retinal edema under different pathological conditions. It has been shown that Müller cells play an important role in K^+^ spatial buffering in the retina which is known as "K^+^ siphoning". In this model, the Müller cells take up the excess extracellular K^+^ ions released during neuronal activity and extrude them into the blood and the vitreous body [10]. The inwardly rectifying potassium channels, particular the subtype of Kir4.1, have been critically implicated in this process [11]. The Kir4.1 is enriched and highly restricted in the endfoot membranes of the Müller cells facing the vitreous body and the retinal blood vessels. It shows rather weaker rectification than that of strong inwardly rectifying K^+^ channels such as Kir2.1, allowing either inward or outward K^+^ currents in dependence on the membrane potential and on the concentration of extracellular K^+^. The polarized distribution and weak rectification enable Kir4.1 to extrude K^+^ from the Müller cell endfeet into blood and vitreous, which is very important for K^+^ siphoning [12]. Recent reports show a decreased expression of Kir4.1 protein in Müller cells during retinal ischemia reperfusion, uveoretinitis, and diabetic retinopathy. The Müller cells in the retinas under these pathological conditions display a downregulation of inward K^+^ currents and a hypotonic cell swelling, which may contribute to the development of cytotoxic macular edema [13-15].

Aquaporin 4 (AQP4) is the most abundant water channel in the brain and retina. It has been suggested to be critically involved in the pathogenesis of brain edema under various injurious circumstances [16,17]. In the retina, AQP4 shows a similar polarized distribution with Kir4.1 in the endfoot membranes of Müller cells [18], which facilitates the water flux at the glio-vascular and glio-vitreal interfaces. The role of AQP4 in retinal edema still remains unclear. It has been shown the deletion of AQP4 in mice significantly reduces retinal edema and preserves the retinal function and architecture after retinal ischemia [19]. However, there is only a slight downregulation of AQP4 expression in the postischemic rat retina, which does not support the idea that retinal edema after ischemia is due to changes in AQP4 expression [13].

EIU is an animal model of human ocular inflammation, and is induced by the administration of lipopolysaccharide (LPS), a component of the Gram-negative bacterial outer membrane [20]. To investigate the roles of Kir4.1 and AQP4 in the development of inflammatory retinal edema, we used the animal model of EIU produced by the intravitreal injection of LPS to analyze the changes in Kir4.1 and AQP4 expression in the retina.

## Methods

### Animal procedures

All experiments were performed on male Wistar rats (6-8 weeks). LPS from Escherichia coli 026:B6 (Sigma, St Louis, MO) was dissolved in sterile phosphate-buffered saline (PBS) at a concentration of 2 mg/ml. Rats (n=78) were anesthetized by intraperitoneal injection of mixture of ketamin (80 mg/kg) and xylazine (10 mg/kg). A single dose of 5 μl of diluted LPS solution was injected intravitreally into one eye of each rat using a Hamilton microinjector (Hamilton Co., Reno, NV) under a dissecting microscope. PBS (5 μl) was administered to the other eye as sham-treated control. Thirteen rats were left untreated. All animals were treated in accordance with the ARVO statement for the Use of Animals in Ophthalmic and Vision Research.

### Clinical and histopathological examinations

Animals were examined with a slit lamp at 6, 12, 24 h, and 3, 7, and 14 days after LPS or PBS injection. The intensity of clinical ocular inflammation was scored on a scale of 0-4 using the scoring system devised by Ruiz-Moreno et al. [21] as follows: 0, no inflammatory reaction; 1, discrete inflammatory reaction; 2, moderate dilation of the iris and conjunctival vessels; 3, intense iridal hyperemia, with flare in the anterior chamber; 4, same clinical signs as 3 plus the presence of fibrinoid exudation in the papillary area, with intense flare in the anterior chamber.

For histopathological examination, the rats were anesthetized, and killed by transcardial perfusion with heparinized isotonic saline, followed by 4% paraformaldehyde in PBS. The eyes were removed and the anterior segments were dissected, postfixed for 2 h in 4% paraformaldehyde, and then rinsed in saline equilibrated through a sucrose gradient (10-30% in PBS), and embedded in OCT compound (Tissue-Tek; Sakura Finetek, Inc., Torrance, CA). Serial frozen sections (10 μm thick) were prepared. Some of the sections were stained with hematoxylin-eosin, and the others were stained immunohistochemically. The sections stained with hematoxylin-eosin were observed with an Olympus BX51 light microscope (Olympus Optical Co., Ltd., Tokyo, Japan). Infiltrating inflammatory cells, including polymorphonuclear neutrophils and mononuclear cells, were observed in the anterior chamber angle, iris-ciliary body stroma, and retina of the eyes of EIU rats. EIU was histopathologically scored using Cousins's classification [22] on the following scale: 0, between zero and nine inflammatory cells within the entire microscopic section, and the absence of other stigmata of inflammation; 1, more than nine inflammatory cells within the entire microscopic section but less than 25 inflammatory cells within any given high-power (40X) field, or at least five inflammatory cell in the entire cross-section accompanied by anterior chamber protein and iris hyperemia; 2, more than 25 but less than 60 inflammatory cells in a high-power field (40X) with the densest concentration of infiltrate; 3, 60 or more inflammatory cells in a single high-power field (40X).

### Immunohistochemistry

For immunohistochemical studies, the sections were blocked with 10% normal goat serum in PBS containing 0.2% Triton X-100 for 1 h at room temperature. The primary antibodies were rabbit anti-Kir4.1 (1:200; Alomone Laboratories, Jerusalem, Israel), rabbit anti-AQP4 (1:250, Chemicon, Temecula, CA), and mouse anti-glial fibrillary acidic protein (GFAP; 1:1000, Transduction Laboratories, Lexington, KY). All were diluted in PBS with 5% goat serum plus 0.1% Triton X-100. After incubation with the primary antibodies overnight at 4 °C, the sections were washed and incubated with Alexa-594-conjugated goat anti-rabbit secondary antibody or Alexa-488-conjugated goat anti-mouse antibody (Molecular Probes, Eugene, OR) at 1:500 for 2 h at room temperature. After the sections had been washed with PBS, they were mounted and observed with a confocal fluorescence microscope (TCS4D, Leica, Wetzlar, Germany).

### Reverse transcriptase polymerase chain reaction

After the rats were killed, the total RNA was extracted from freshly dissected sensory retinas using the TRIzol® Total RNA Isolation System (Invitrogen, Carlsbad, CA, USA). The total RNA was quantified by ultraviolet spectrophotometry by measuring OD_260_ and OD_280_. To eliminate contamination with genomic DNA, the RNA samples (1 μg) were treated with DNaseI (deoxyribonuclease I, amplification grade; Invitrogen, Life Technologies, Inc., Gaithersburg, ML) and then subjected to reverse transcription using the RevertAid H Minus First Strand cDNA Synthesis Kit (Fermentas, Life Science, M-Medical Srl, Cornaredo, Italy). An aliquot (1 μl) of each cDNA sample was amplified with forward and reverse primers with the following sequences. For Kir4.1 mRNA (GenBank accession no. NM_031602), 5'-GTA GAC ACA GCC TCT GAT AGC C-3' (836-857) and 5'-AGC AGG TGT GAA CTC GTA GC-3' (1060-1041), generating a 225-bp amplicon. Reactions were performed for 30 cycles (94 °C, 45 s; 58 °C, 30 s; 72 °C, 60 s). For AQP4 mRNA (GenBank accession number U14007), 5'-GGG TTG GAC CAA TCA TAG GCG CT-3' (701-723) and 5'-GCA GGA AAT CTG AGG CCA GTT CTA GG-3' (1030-1005), generating a 330-bp amplicon. Reactions were performed for 25 cycles (94 °C, 30 s; 60 °C, 30 s; 72 °C, 60 s). For β-actin mRNA (GenBank accession number NM_031144), 5'-TAA AGA CCT CTA TGC CAA CAC AGT-3' (951-974) and 5'-CAC GAT GGA GGG CCG GAC TCA TC-3' (1191-1168), generating a 240-bp amplicon. Reactions were performed for 25 cycles (95 °C, 30 s; 60 °C, 30 s; 72 °C, 60 s). All reactions were performed within the linear range of amplification and β-actin was used as the control. The nucleotide sequences of the PCR products were confirmed by direct sequencing analysis. After amplification, the PCR products (5 μl) were run on a 2.0% agarose gel, stained with ethidium bromide, and photographed. The images were quantitated using the software ImageJ (NIH).

### Western blotting analysis

At different time points, the rats were killed with an overdose injection of pentobarbital and their eyeballs were enucleated. The neural retinas dissected from the eyecups were weighed and homogenized in ice-cold lysis buffer containing 50 mM Tris-HCl (pH 7.4), 250 mM NaCl, and 1% Nonidet P-40, with a protease inhibitor cocktail. The protein concentrations of the samples were measured using the Lowry method [23]. Each protein sample (30 μg) was separated by 12% sodium dodecyl sulfate polyacrylamide gel electrophoresis (SDS-PAGE) for AQP4 and by 7.5% SDS-PAGE for Kir4.1, and then transferred onto polyvinylidene difluoride (PVDF) membranes. Immunoblotting was performed using rabbit anti-Kir4.1 (1:1000) or rabbit anti-AQP4 (1:1000) as the primary antibodies and horseradish-peroxidase-conjugated IgG as the secondary antibody. The reaction products were visualized with ECL Western blotting detection reagents (Amersham, Buckinghamshire, UK). Images were captured with a Fuji Film LAS3000 imaging system and analyzed with MultiGauge software (Fuji Film, Tokyo, Japan).

### Statistical analysis

Quantitative data were expressed as means±SEM, and were analyzed with one-way analysis of variance (ANOVA) followed by Dunnett's test for multiple comparisons among experimental groups with control groups. Statistical analysis was performed using the SPSS 10 statistical software. The results were considered statistically different at p<0.05.

## Results

### Clinical and histopathological scoring of endotoxin-induced uveitis

**Figure 1:**
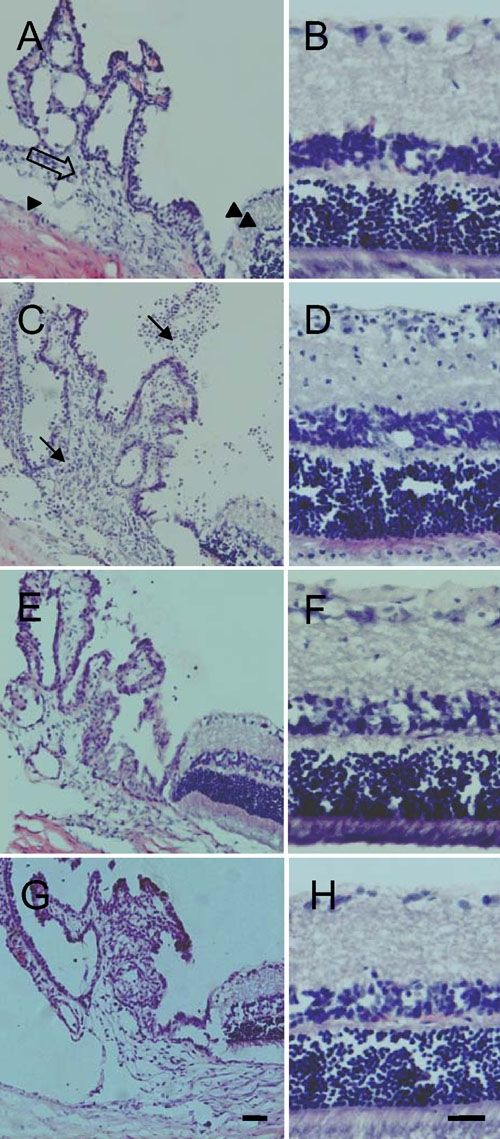
Hematoxylin-eosin stained eyes. **A**, **B**: No inflammatory cells were seen in the anterior chamber (arrowhead), iris-ciliary body (blank arrow), posterior chamber and retina (double arrowhead) of untreated control eyes. **C**, **D**: Inflammatory cells (arrows) were seen in the anterior chamber, iris-ciliary body, posterior chamber and retina one day after lipopolysaccharide (LPS) injection. **E**, **F**: Inflammation had almost subsided and only a few inflammatory cells remained seven days after LPS injection. **G**, **H**: No inflammatory cells were seen in the sections one day after phosphate-buffered saline treatment. Scale bar represents 20 μm.

Uveitis developed quickly after the intravitreal LPS injection. Ocular changes, such as dilation of the iris and conjunctival vessels, iridal hyperemia, and cells and flare in the anterior chamber, were observed as early as 6 h after LPS injection. These signs peaked at 24 h, then subsided gradually during the following period, and had nearly disappeared by day 7. EIU clinical grades were 1.9 at 6 h, 3.7 at 12 h, 4 at 1 day, 2.5 at 3 day, 0.3 at 7 day, and 0 at 14 day (Table 1). The eyes of the rats were enucleated at 6, 12, 24 h, and 3, 7, and 14 days after the intravitreal injection of LPS or PBS and stained with hematoxylin-eosin. Compared with the no-treatment eyes (Figure 1A,B), some inflammatory cells appeared in the anterior chamber and posterior chamber as early as 6 h after the LPS injection. At 12 h after the intravitreal injection of LPS, inflammatory cells appeared in the stroma of the iris-ciliary body and the retina, as well as the anterior chamber, posterior chamber, and vitreous body. These changes became more intense and reached a peak 24 h after the injection (Figure 1C,D). At three day, inflammation became weak and the number of inflammatory cells had decreased. Seven days after the intravitreal injection of LPS, only rare inflammatory cells were detected in the iris-ciliary body and retina (Figure 1E,F). Fourteen days after the injection, the retina had recovered from inflammation and no inflammatory cells were seen in the sections. The histopathological grades were 1.3 at 6 h, 2.8 at 12 h, 3 at 1 day, 2.3 at 3 day, 0.5 at 7 day, and 0 at 14 day (Table 1). In our experiments, the histopathological findings correlated well with the clinical signs. There was no clinical or histopathological evidence of uveitis in the PBS-treated rats (Figure 1G,H).

**Table 1:** Clinical and histopathological scoring of endotoxin-induced uveitis.

**Time** **(after LPS injection)**	**Clinical grades** **(mean±SEM, n=10)**	**Histopathological grades** **(mean±SEM, n=4)**
0 h	0	0
6 h	1.9±0.2	1.3±0.2
12 h	3.7±0.1	2.8±0.3
24 h	4.0±0	3.0±0
3 d	2.5±0.2	2.3±0.2
7 d	0.3±0.1	0.5±0.2
14 d	0	0

The clinical signs of endotoxin-induced uveitis (EIU) were examined with a slit lamp and graded on a scale from 0 (normal) to 4 (severe). The hematoxylin-eosin-stained sections were observed under an optical microscope and scored ranging from 0 (normal) to 3 (severe).

### Immunoreactivity of Kir4.1, aquaporin-4, and anti-glial fibrillary acidic protein in retina

**Figure 2:**
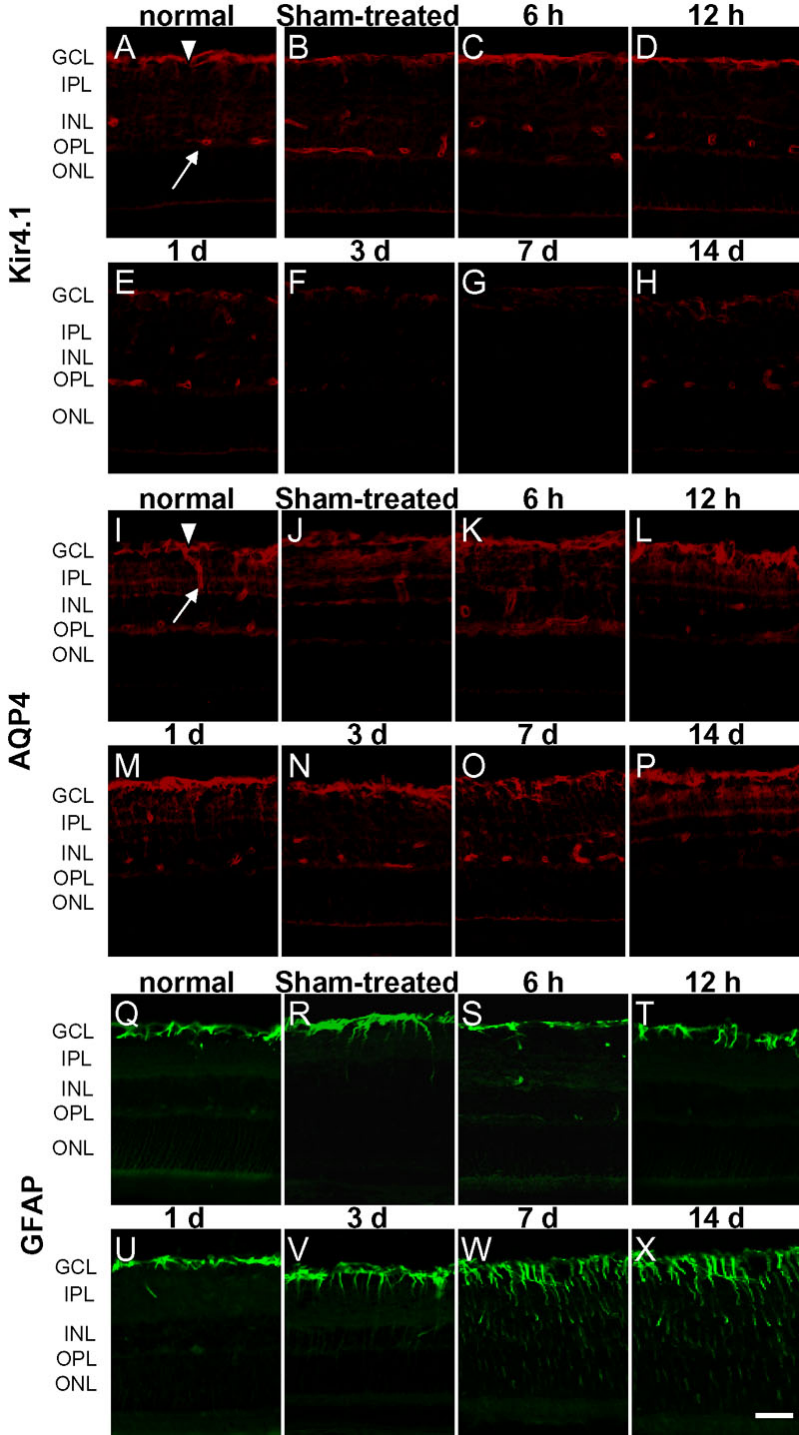
Immunohistochemical detection of Kir4.1, aquaporin-4, and anti-glial fibrillary acidic protein in the retina. In the normal eyes, Kir4.1 (**A**) and aquaporin-4 (AQP4; **I**) were enriched in the endfoot membranes facing the vitreous body (arrowheads) and retinal blood vessels (arrows). Staining for AQP4 (**K**-**P**) maintained the same pattern during the different stages of endotoxin-induced uveitis (EIU), and only a slight reduction in immunostaining was seen in the inner plexiform at 1-7 day after lipopolysaccharide (LPS) injection. Kir4.1 (**C**-**H**) immunoreactivity decreased significantly from one day after LPS injection, had almost disappeared at 3-7 day after injection, and had partially recovered by 14 days. Anti-glial fibrillary acidic protein (GFAP; **Q**-**X**) was predominantly found in astrocytes in the retinas of the untreated controls. Seven days and 14 days after intravitreal LPS injection, GFAP (**W**-**X**) immunoreactivity was significantly increased in Müller cells. In the retinas of sham-treated eyes, the immunostaining for Kir4.1 (**A**-**H**) was unchanged at 3 day after phosphate-buffered saline (PBS) treatment (**B**). AQP4 immunoreactivity was unchanged at one day after PBS treatment (**J**). GFAP immunoreactivity was mildly increased at 7 day after PBS treatment (**R**). GCL indicates ganglion cell layer; INL indicates inner nuclear layer; IPL indicates inner plexiform layer; ONL indicates outer nuclear layer; OPL indicates outer plexiform layer. Scale bar represents 20 μm.

Immunostaining for Kir4.1, AQP4, and GFAP was performed at various time points after LPS or PBS injection. Kir4.1 and AQP4 showed the same polarized distribution patterns in the retina and retinal blood vessels. They were enriched in the endfoot membranes facing the vitreous body and retinal blood vessels (Figure 2A,I). The Kir4.1 immunoreactivity decreased significantly from one day after LPS injection, had almost disappeared at 3-7 days after injection, and had partially recovered by 14 days (Figure 2C-H). The immunostaining for AQP4 maintained the same pattern during the different stages of EIU and only a slight reduction in immunoreactivity was observed in the inner plexiform from 1-7 days after LPS injection (Figure 2K-P). GFAP, a cellular marker for reactive gliosis of the Müller cells, was predominantly found in astrocytes in the retinas of untreated control rats. At 7 days and 14 days after intravitreal LPS injection, the GFAP immunoreactivity in the Müller cells was significantly increased (Figure 2Q-X). A mild increase in GFAP immunoreactivity was also observed in the Müller cells at 7 days (Figure 2R) and 14 days after PBS treatment. No obvious changes in immunostaining for Kir4.1 (Figure 2B) or AQP4 (Figure 2J) were seen in the retinas of PBS-treated rats.

### Kir4.1 and aquaporin-4 mRNA expression in endotoxin-induced uveitis retinas

**Figure 3:**
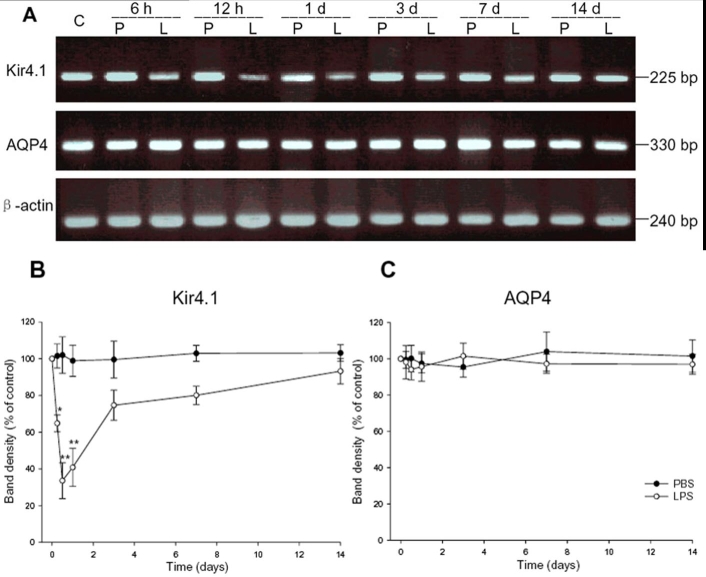
Time course of Kir4.1 and aquaporin-4 mRNA expression in lipopolysaccharide- or phosphate-buffered saline-treated rats. **A**: Total RNA (1 μg) was used for reverse transcriptase polymerase chain reaction (RT-PCR). A 330-bp product for aquaporin-4 (AQP4), a 225-bp product for Kir4.1, and a 240-bp product for β-actin were separated on a 2.0% agarose gel. **B**, **C**: The relative levels of Kir4.1 and AQP4 mRNA expression were quantified. Compared with the control, there was a significant decline in Kir4.1 in lipopolysaccharide (LPS)-treated animals, whereas there was no change in AQP4 after LPS injection (means±SEM, n=4; asterisk (*) indicates p<0.05, double asterisks (**) signifies p<0.001 versus control). C indicates control; P indicates phosphate-buffered saline; L represents lipopolysaccharide.

The relative levels of Kir4.1 and AQP4 mRNA expression during EIU were measured by semiquantitative RT-PCR experiments and subsequently analyzed after normalization to those of β-actin (Figure 3). The mean values for the untreated control eyes were set at 100%. In the early stages of EIU, the expression of Kir4.1 mRNA decreased sharply, reaching a minimum at 12 h (31%, p<0.001). It then increased gradually and had partially recovered 14 days after LPS injection (92%, p>0.05; Figure 3B). In contrast, AQP4 mRNA remained unchanged after LPS treatment (Figure 3C). In PBS-treated rats, no obvious changes were observed in either Kir4.1 or AQP4 mRNA expression at various time points.

### Kir4.1 and aquaporin-4 protein expression in endotoxin-induced uveitis retinas

**Figure 4:**
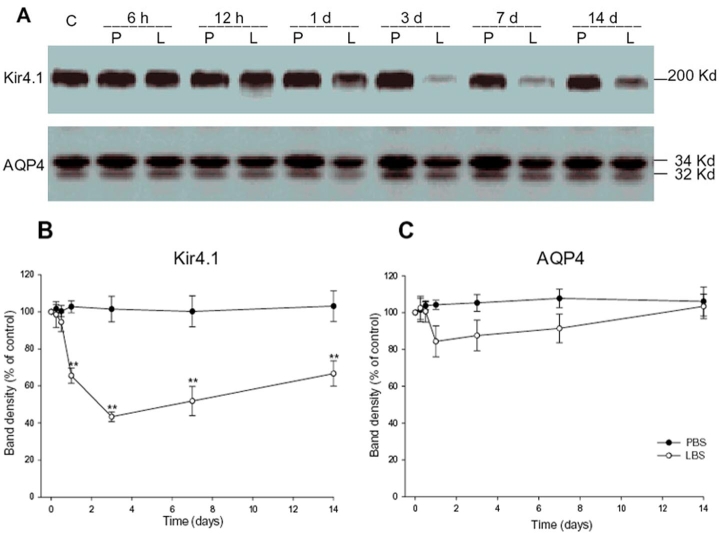
Time courses of Kir4.1 and aquaporin-4 protein expression in retinas from lipopolysaccharide- or phosphate-buffered saline-treated rats. **A**: Equal amounts of protein (30 μg) were subjected to immunoblotting analysis. A band at about 200 kDa represents Kir4.1 in its tetrameric form; aquaporin-4 (AQP4) consists of two bands, representing its M1 and M23 forms. **B**, **C**: The relative levels of Kir4.1 and AQP4 protein expression were quantified. Compared with that of the control, the expression of Kir4.1 was significantly reduced after LPS injection. In contrast, there was only a slight, statistically insignificant decline in AQP4 expression from 1 day to 7 day after LPS injection. (means±SEM, n=5; double asterisks (**) p<0.001 versus control). C indicates control; P indicates phosphate-buffered saline; L indicates lipopolysaccharide.

Immunoblotting for Kir4.1 showed a band at about 200 kDa corresponding to the tetrameric form of the protein, which is in agreement with the results of prior study (Figure 4A) [24]. This band decreased significantly, reaching a minimum at 3 days after the LPS injection (43%, p<0.001, n=5, mean values for the untreated control eyes were set at 100%). Thereafter, it increased slightly but was maintained at a low level until 14 days after LPS injection (64%, p<0.001; Figure 4B). Immunoblotting for AQP4 produced two bands, which represent the two splice variants of AQP4, M1-AQP4, and M23-AQP4 (Figure 4A) [25]. The magnitude of AQP4 expression was assessed by combining the absorbance for both the M1 and M23 forms, as measured by densitometry. The expression of AQP4 was slightly reduced at one day (82%, p>0.05) after LPS injection. It then increased gradually and had nearly recovered to the basal level at 14 days (Figure 4C). In PBS-treated rats, no obvious changes were observed in either Kir4.1 or AQP4 protein expression at various time points (Figure 4B,C).

## Discussion

Several studies have suggested that there exists a tight coupling between water transport and K^+^ clearance in the brain. In the hippocampal slices from α-syntrophin-null mice, the K^+^ clearance was significantly delayed, probably resulting from the mislocalization of AQP4 [26]. It was proposed that a concomitant water flux through perivascular AQP4 is required to sustain the efficient removal of K^+^ after neuronal activation. Another piece of evidence for the functional coupling of water transport and K^+^ clearance is that the stimulus-induced extracellular K^+^ elevation in sensorimotor cortex causes cell swelling accompanied by shrinkage of the extracellular space [27]. In the normal retina, Kir4.1 and AQP4 strictly colocalize in the endfoot membranes of the Müller cell facing the vitreous body and the retinal blood vessels, suggesting that AQP4-mediated water transport by Müller cells is coupled to Kir4.1-mediated K^+^ siphoning [18]. We have shown here that the expression of Kir4.1 was significantly reduced, whereas that of AQP4 was only slightly reduced in retinas after an intravitreal LPS injection. This differential expression of Kir4.1 and AQP4 in the retina suggests that water transport becomes uncoupled from K^+^ siphoning. Since the Kir4.1 channel is predominantly expressed in membranes through which K^+^ ions flow out of Müller cells and enter "extracellular sinks" [12], the downregulation of Kir4.1 may disturb the efflux of K^+^ from the endfeet into blood vessels and vitreous body, followed in turn by an accumulation of K^+^ within the Müller cells. Subsequently, the increased intracellular K^+^ causes an osmotic gradient which drives water from the blood and vitreous into Müller cells, via the AQP4 in the endfeet which is almost unchanged, leads to a swelling of the Müller cells (cytotoxic edema). This hypothesis was also supported by a recent experiment, which showed that Müller cells in retinas from LPS-treated eyes displayed a hypotonic cell swelling, and a downregulation of inward K^+^ currents associated with a decreased expression of Kir4.1 protein in retina slices [14]. Moreover, we assumed that the decreased K^+^ efflux might parallel a decreased water efflux to blood and vitreous, which may impair the absorption of vasogenic edema during EIU.

It has been shown that Kir4.1 and AQP4 share a similar polarized distribution and cocluster in Müller cell membranes by association with the multiprotein dystrophin-glycoprotein complex (DGC) [28]. Meanwhile, recent studies also suggested that the Kir4.1 and AQP4 have different clustering mechanisms and are regulated independently under different pathological conditions. For example, in the α-syntrophin-deficient mouse, AQP4 is largely mislocalized from the endfoot membranes of the Müller cells, whereas Kir4.1 localization is unaffected [29]. This finding suggests that α-syntrophin is partly responsible for anchoring AQP4 but is not involved in anchoring Kir4.1 in the glial endfoot membranes. The different redistribution patterns of Kir4.1 and AQP4 in low- and high-grade human brain tumors also supports the hypothesis of separated mechanisms for the clustering of Kir4.1 and AQP4 [30]. Our experiment shows the mislocation of Kir4.1 in the endfoot membranes of Müller cells and an unchanged AQP4 localization in EIU. This differential expression pattern implies different regulatory mechanisms for Kir4.1 and AQP4 in ocular inflammation, which might be due to their different clustering mechanisms. In addition, the change of Kir4.1 mRNA was several hours earlier than that of Kir4.1 protein, suggesting that the decrease in the synthesis and/or degradation of Kir4.1 mRNA proceeded faster than that of Kir4.1 protein. On the other hand, there was a slight reduction in AQP4 protein level without a marked change in mRNA level, which might suggest that the downregulation of AQP4 protein was caused by decreased synthesis of AQP4 protein or accelerated protein degradation during inflammation.

In vitro experiments have shown that an increase in the extracellular K^+^ concentration upregulates LPS-induced nitric oxide (NO) production in astrocytes [31] but inhibits tumor necrosis factor a (TNF-α) and IL-6 production in activated astrocytes [32]. Moreover, TNF-α causes a marked reduction in inwardly rectifying K^+^ currents in cultured astrocytes [33]. These results suggest that there might be a complicated interaction between potassium ions and inflammatory mediators in the brain. It is known that EIU is accompanied by a subsequent release of proinflammatory cytokines, prostaglandins, NO, and arachidonic acid metabolites in the uvea and retina [34,35]. In our experiments, the expression of Kir4.1 in the retina decreased significantly in the early stages of EIU and the reduction persisted for a long time, even when the inflammation had subsided. Because the changes in Kir4.1 expression and the intensity of ocular inflammation were not in parallel, the role of Kir4.1 in the inflammatory process of EIU requires further investigation.

In summary, our results show the differential expression of Kir4.1 and AQP4 in the retina during EIU in rats, which implies an uncoupling of AQP4-mediated water transport from Kir4.1-mediated K^+^ siphoning. This may result in a disturbance of potassium and water homeostasis in the retina, which in turn leads to Müller cells swelling (cytotoxic edema) and impairment of the absorption of vasogenic edema during EIU. Moreover, the differential expression of Kir4.1 and AQP4 implies different clustering mechanisms of Kir4.1 and AQP4 in the endfoot membranes of Müller cells. The disturbed potassium homeostasis might be involved in the inflammatory process in the retina by interacting with the inflammatory mediators released during EIU. Further investigation of these mechanisms may lead to a new understanding of EIU, as well as to novel concepts for the therapeutic management of retinal edema.
